# State-of-the-Art Molecular Genetics and Genomics in Germany

**DOI:** 10.3390/ijms241814096

**Published:** 2023-09-14

**Authors:** Jan Lukas

**Affiliations:** 1 Translational Neurodegeneration Section “Albrecht-Kossel”, Department of Neurology, University Medical Center Rostock, 18147 Rostock, Germany; jan.lukas@med.uni-rostock.de; 2 Center for Transdisciplinary Neurosciences Rostock (CTNR), University Medical Center Rostock, University of Rostock, 18147 Rostock, Germany

The Special Issue State-of-the-Art Molecular Genetics and Genomics in Germany focuses on German researchers and their international peers, covering their recent advances in genetics, genomics, epigenetics, and cytogenetics/cytogenomics in relation to prokaryotic and eukaryotic multicellular to mammalian organisms in arras ranging from basic to medical research. The resulting compilation of articles thus covers a broad range from plants to humans dealing with evolutionary and pathogenic processes and mechanisms and technological advancements leading to translational progress in molecular forensics, diagnostics and novel therapeutic approaches.

Researchers are constantly developing new DNA and RNA sequencing platforms to do justice to the variety of complex questions and demands involved in the medical context in order to provide faster, more convenient and cost-effective solutions. Since its inception in the 1970s, this technology has experienced considerable progress. Short tandem repeat (STR) analysis is usually performed with PCR amplification followed by capillary electrophoresis (CE), recently combined with the high-throughput format using next generation sequencing (NGS) techniques. However, commercial kits are only profitable when applied to high numbers of samples. Furthermore, problems of underperformance arise with degraded or inhibitor-contaminated samples. Poethe et al. [1] present an adapted protocol based on a shallow sequence (lower pass) output (called maSTR assay) and a new bioinformatics pipeline (called SNipSTR) to flexibly investigate genome-wide genetic variation for the purpose of molecular forensics and diagnostics. 

The presence of post-replicative DNA modification is widespread in both prokaryotic and eukaryotic organisms. The process of DNA methylation utilizes the enzymatic addition of methyl groups to the nucleobases adenine and cytosine enabling the development, differentiation, and maintenance of cellular identity in mammals by means of gene expression control without requiring irreversible genomic alterations. Riemens et al. [2] developed a novel method based on a combination of laser capture microdissection (LCM) and limiting dilution bisulfite pyrosequencing (LDBSP) to obtain information on the methylation status of single alleles using a pool of fifty subtype marker-positive neurons from unfixed postmortem human brains as an example. In combination with a novel data analysis pipeline, the authors claim that the technology allows the safe use of data indicative of the presence of two or three alleles represented in the PCR reads that were typically excluded in previous studies using single-cell bisulfite sequencing techniques “inducing bias or […] reducing or reinforcing effect sizes”. This alternative strategy is suitable for the study of DNA methylation patterns in the human brain, for example, for the detection of psychiatric or neurodegenerative diseases.

A different enhancement of the sequencing technology was also addressed by Skowronek et al. [3] who developed a Cas9 mediated approach to analyze specific gene regions of larger scale. The group addressed the challenges of sequencing-based molecular diagnostics in cerebral cavernous malformations (CCM), which occur in sporadic and familial forms. A significant proportion of the pathogenic variants of familial CCM are due to large deletions. Hence, previous diagnostic techniques based on multiplex ligation-dependent probe amplification (MLPA) and short-read sequencing had their limitations. A Cas9-linked technology using specific crRNA probes enabling subsequent real-time long-read nanosequencing had been intended to remedy the situation. It enabled the identification of mutation breakpoints in the disease-associated genes *CCM1*, *CCM2*, and *CCM3*. This new PCR-based assay might especially be efficient for familial pathologies inherited on mutations within repetitive DNA sequences.

Amberger and colleagues bring up another application for CRISPR/Cas9 technology here, which focuses on reducing the cell biological side effects of gene and cell therapies to render these techniques reliably safe [4]. The authors constructed an inducible cell killing system (“suicide switch”) to reduce unwanted risks such as inflammation, neurotoxicity, and tumorigenic potential of, e.g., grafted genetically engineered cells. This complex system involves a combination of a transcriptionally and post-translationally pharmacologically regulatable Cas9 enzyme coupled with repetitive Alu retrotransposon-specific single-guide RNAs (sgRNAs) that leads to irreparable genome fragmentation and subsequent cell death through Cas9-induced double-strand breaks (DSBs). The suicide switch construct can be used in the context of gene and cell therapies as a co-administration alongside therapeutic gene delivery to allow for emergency intervention in case of the malignant transformation of the grafts.

A mechanistic study of the lipid phosphatase encoded by the *PTEN* gene and known tumor suppressor is provided by Pei and colleagues [5]. The study provides strong evidence for the involvement of PTEN in homologous recombination (HR), the error-free arm of DNA damage repair of DSBs. The study designed a PTEN deletion model in glioblastoma cells and demonstrated radiosensitization of tumor cells under PTEN knockdown conditions and a mechanistic switch to error-prone DSB processing, while simultaneously reducing RAD51, a key regulator of HR. The data suggested that PTEN status can be considered a potential indicator of the effectiveness of certain tumor treatment options such as ionizing radiation (IR) and poly-ADP-ribose polymerase inhibitors (PARPi). Although normal cells also underwent radiosensitization after PTEN knockdown, a pharmacological intervention strategy using PARPi can usefully assist the treatment design through the highly localized application of IR.

Coming to X-linked rare diseases. As reported by Alfen et al., for a number of exonic mutations in the *GLA* gene, previously linked to missense enzyme variants in Fabry disease, abnormal splicing events could be detected [6]. The molecular pathomechanism of the several hundred exonic *GLA* point mutations is becoming increasingly important, as both prediction of disease severity and treatability of patients with the approved pharmacological chaperone rely on an in vitro cellular enzyme activity assay that is unable to screen for splicing aberrations. Minigene splicing reporter assay analyses detected abnormal splicing for mutations at exon/intron boundaries as well as for a mutation located further intra-exonically indicating a potential misclassification of a larger number of mutations. As successfully applied to Fabry disease, Schneider et al. [7] are also pursuing replacement of the defective protein as a therapeutic approach for the rare X-linked hypohidrotic ectodermal dysplasia (XLHED). XLHED is a developmental disorder of the ectodermal derivatives that is associated with the inability to sweat and carries the risk of severe hyperthermia. A small cohort of nine patients have been administered recombinant EDA internalized via the Fc receptor postnatally or via intra-amniotic administration since 2013 and long-term study results are presented here. 80% of the XLHED patients are anhidrotic and are heat intolerant. Thus, the study made use of perspiration and body temperature as targeted outcomes. The patients seemed to be amenable to timely EDA1 replacement therapy as performed in the study. Administration of Fc-EDA1 doses in utero prevented potentially life-threatening hyperthermia and febrile convulsions in the children within the first year of life. Furthermore, the results of the study indicated sweat gland function in long-term follow-up up to six years after administration of the protein. 

In the study by Paetzold et al. [8], the topic of transcriptome evolution in *Ranunculus auricomus* is traced in relation to sexual and asexual seed formation. Asexual seed formation (apomixis) seems to be generally preceded by hybridization of the parent species. Hence, the phylogenetic hybridization networks that may establish apospory as a heritable trait are currently not known. Therefore, the researchers examined transcriptomes of early-generation hybrids of *R. auricomus* to track the shift from sexual to asexual seed formation during the reproductive process. For this purpose, synthetic aposporous F2 hybrid generation samples were compared with parental samples. The genes and gene networks subjected to diversifying selection contribute to the explanation of a heritable regulatory mechanism as a major driving force for the switch and can serve as potential candidates for apomictic model systems.

This Special Issue provides advanced technical and mechanistic insights into disease mechanisms, diagnosis, and therapy in genetic diseases and sexual and asexual plant reproduction. Among other topics, various DNA analysis tools, the application of a suicide switch for safer gene and cell therapies, and new insights into the role of PTEN, a dephosphorylating enzyme of signal transducing molecules such as AKT1, PIP₂ and PIP₃, in error-free DSB processing are presented. 
